# Heterogeneity and plasticity of epidermal stem cells

**DOI:** 10.1242/dev.104588

**Published:** 2014-07

**Authors:** Troels Schepeler, Mahalia E. Page, Kim B. Jensen

**Affiliations:** 1 BRIC - Biotech Research and Innovation Centre, University of Copenhagen, Ole Maaløes Vej 5, Copenhagen N DK-2200, Denmark; 2 Wellcome Trust & Medical Research Council Cambridge Stem Cell Institute, Tennis Court Road, Cambridge CB2 1QR, UK

**Keywords:** Stem cells, Epidermis, Regeneration, Tissue homeostasis

## Abstract

The epidermis is an integral part of our largest organ, the skin, and protects us against the hostile environment. It is a highly dynamic tissue that, during normal steady-state conditions, undergoes constant turnover. Multiple stem cell populations residing in autonomously maintained compartments facilitate this task. In this Review, we discuss stem cell behaviour during normal tissue homeostasis, regeneration and disease within the pilosebaceous unit, an integral structure of the epidermis that is responsible for hair growth and lubrication of the epithelium. We provide an up-to-date view of the pilosebaceous unit, encompassing the heterogeneity and plasticity of multiple discrete stem cell populations that are strongly influenced by external cues to maintain their identity and function.

## Introduction

The skin is the largest organ of the body and consists of multiple layers with distinct developmental germ layer origins. The epidermis forms the outermost, water-impermeable layer that protects against the hostile environment and retains bodily fluids inside. It is composed of epithelial cells (both keratinocytes and Merkel cells) in addition to small populations of Langerhans cells, gamma delta (γ∂) T-cells and melanocytes. The dermis underneath the epidermis forms the basis for complex interactions with fibroblasts, endothelial, neuronal, muscle and immune cells and provides a three-dimensional framework that supports the maintenance of the epidermis.

**Fig. 1.**
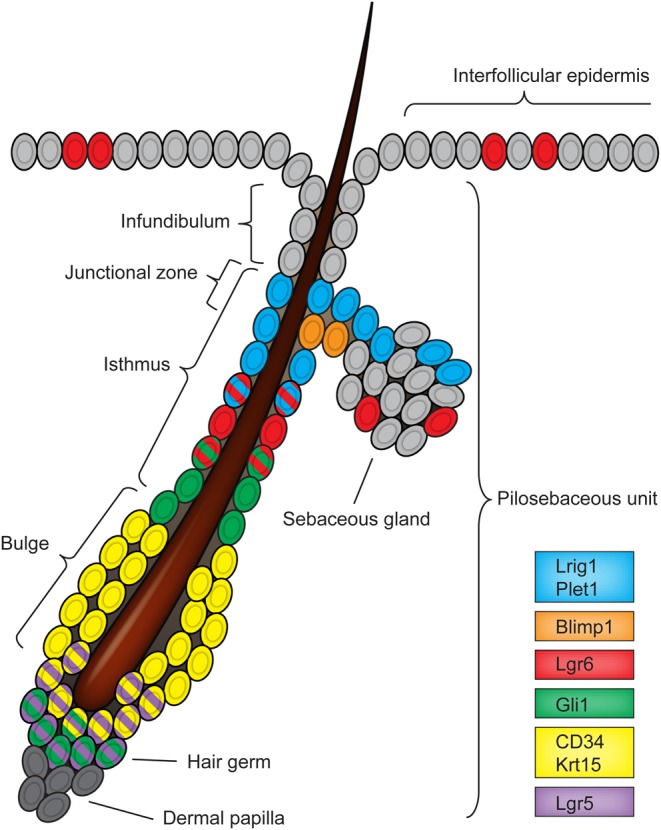
**Hair follicle stem cell compartments.** Different stem cell populations are shown in the mouse resting (telogen) adult hair follicle. Each stem cell compartment is defined by distinct protein expression and gene promoter activity (see key); cells with multiple colours express multiple markers.

The main component of the epidermis is the interfollicular epidermis (IFE), which forms the protective barrier against the outside environment. The IFE is a stratified epithelium in which proliferating cells are anchored to the basement membrane closest to the dermis. As cells lose affinity for the basement membrane, they initiate terminal differentiation and, following extensive remodelling of intracellular proteins, intercellular junctions, lipid extrusion and nuclear fragmentation, the cells eventually become highly cross-linked scales that are exfoliated from the surface of the skin. The pilosebaceous unit (PSU) is a prominent structure associated with the IFE (Fig. 1). It has important functions within the tissue that are mediated via its components: infundibulum, isthmus, sebaceous glands and the hair follicle. The infundibulum forms the upper part of the PSU between the IFE and the isthmus. The isthmus forms the mid-region starting at the top of the bulge and ending at the infundibulum, where it segregates hair follicle and interfollicular differentiation markers and creates a funnel for the hair shaft (Fig. 1). The upper region of the isthmus adjacent to the infundibulum and the sebaceous gland is defined as the junctional zone (JZ) (Jensen et al., 2009). The sebaceous gland produces sebum, an antiseptic oily substance that lubricates the hair and the surface of the skin. Similar to the IFE, proliferating cells in the sebaceous gland anchored to the basement membrane support turnover of differentiated cells as they burst and release their lipid content. The lower, permanent PSU can be divided into two parts: the bulge and the hair germ. Hair follicle stem cells reside in the bulge, whereas the hair germ is located directly above the dermal papilla and forms the germinal centre for hair follicle growth (Fig. 1) (Silver and Chase, 1977).

In mice, PSU size varies depending on the auxiliary functions: large sensory vibrissae (whiskers) originate from a large PSU, whereas the smaller zig-zag hairs that make up the heat-insulating coat tend to have a correspondingly small PSU. Despite these variations, the overall composition of the PSU is the same (Sundberg and Hogan, 1994). The lower, permanent part of the PSU supports successive rounds of hair growth, which continues throughout life in three distinct phases: anagen, catagen and telogen. Depending on the mouse strain and body site, the initial phase of PSU morphogenesis ceases at postnatal day (P) 14-18, from which point hair cycling is initiated by regression of the hair structure, known as the catagen phase. During this process, the bottom of the hair follicle undergoes rapid apoptosis. After a short resting phase of 3-4 days known as the telogen phase, hair growth is reinitiated in anagen phase and the cycle continues. Hair cycling through the three phases continues throughout life; however, it is only the first postnatal cycle that is uniformly synchronised in the mouse. This ends with another catagen phase and a long telogen phase at ∼P42-49, which lasts 2-3 weeks (Alonso and Fuchs, 2006). Subsequent hair cycles of individual follicles are then asynchronous and are instructed by local signals (Plikus et al., 2008). The cyclic nature of this process is governed by an intricate interplay between dermal cells and epidermal keratinocytes in the lower PSU (Fig. 1) (Plikus et al., 2008; Driskell et al., 2009; Greco et al., 2009).

The distinct components of the epidermis are maintained throughout life by resident stem cells. In humans, the first seminal experiments suggesting the existence of epidermal stem cells came from *in vitro* cultures, in which epithelial cells from small skin biopsies were serially propagated and shown to form stratified squamous epithelium with more advanced keratinisation of upper cell layers (Rheinwald and Green, 1975). Stem cell behaviour was proven by the successful engraftment to, and long-term maintenance of, cultured keratinocytes in burns victims (Gallico et al., 1984). In general, a high degree of cellular heterogeneity defined by marker expression, cell division rate and ultrastructure, has been observed both within the basal layer of the human IFE (Jones et al., 1995; Li et al., 1998; Jensen et al., 1999) and in the PSU (Cotsarelis et al., 1990; Rochat et al., 1994; Lyle et al., 1998; Ohyama et al., 2006). These observations led to the proposal that stem cells exist within distinct niches and that these cells can give rise to progeny with limited proliferative potential, also known as transit amplifying cells. Similar observations have been made for the mouse epidermis, which will be the focus of this Review.

The prevailing model for epidermal maintenance places multipotent stem cells at the apex of a cellular hierarchy. This is based on a combination of cell culture, lineage-tracing and transplantation studies (Jaks et al., 2008; Snippert et al., 2010; Blanpain et al., 2004; Claudinot et al., 2005; Jensen et al., 2008). However, it is not clear whether transplantation studies provide a true reflection of multipotency during steady-state homeostasis and, furthermore, the exact location of the multipotent stem cells remains unclear. Recent data from live-imaging studies and long-term fate-mapping experiments have demonstrated regionally restricted contributions from multiple distinct stem cell niches in the PSU during homeostasis (Ghazizadeh and Taichman, 2001; Morris et al., 2004; Levy et al., 2005; Jaks et al., 2008; Brownell et al., 2011; Page et al., 2013). Furthermore, transplantation and injury studies demonstrate that such regional restriction of discrete stem cell populations breaks down after tissue damage, as stem cells have been observed to regenerate all structures of the epidermis under such conditions (Levy et al., 2005, 2007; Nowak et al., 2008; Jensen et al., 2009; Brownell et al., 2011; Page et al., 2013). This forms the basis for an updated model of tissue maintenance, which is governed by a number of equipotent stem cell populations with discrete functions during homeostasis. In this Review, we will discuss the basis for this model and its functional relevance.

## The emergence of cellular heterogeneity within the PSU

**Fig. 2.**
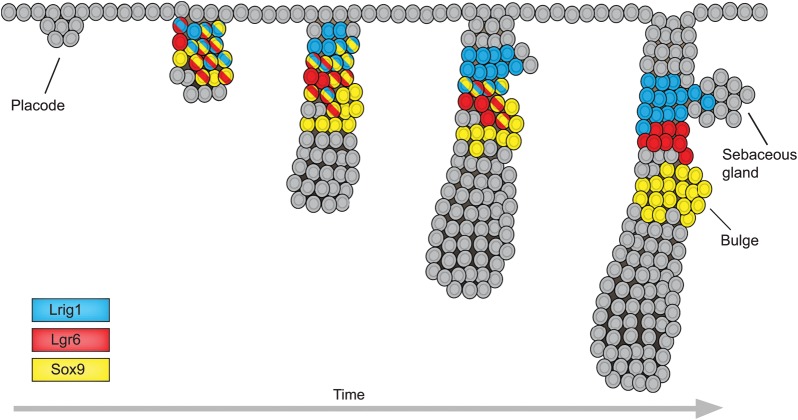
**Emergence of distinct stem cell populations during morphogenesis of the pilosebaceous unit.** During development, pilosebaceous formation is initiated from an early epidermal structure (the placode) that develops into a fully formed pilosebaceous unit (PSU) through a series of steps involving complex interactions with existing dermal cells. Initially, various stem cell markers are co-expressed within the same region of the developing PSU, but at later stages marker expression is associated with segregation of cells into distinct domains. Cells with multiple colours express multiple markers.

The epidermis forms as a flat single-layered epithelium from the surface ectoderm. The appearance of PSUs proceeds in waves depending on the associated hair type, starting with whisker follicles, then awl/auchene follicles and lastly zig-zag hairs. Although the size of the PSU varies between the different hair types, they all follow essentially the same morphological transitions (reviewed by Schmidt-Ullrich and Paus, 2005). Focal elevation in Wnt signalling initiates PSU formation and the growing structure subsequently extends into the underlying mesenchyme (Gat et al., 1998; St-Jacques et al., 1998; Huelsken et al., 2001). Analysis of the developing PSU demonstrates co-expression of the future adult stem cell markers Sox9, Lgr6 and Lrig1 (Nowak et al., 2008; Jensen et al., 2009; Snippert et al., 2010; Frances and Niemann, 2012). As the PSU extends further into the dermis, expression of these stem cell markers segregates into distinct domains. These include a quiescent region that is positive for future bulge stem cell markers, such as Sox9, Nfatc1 and Tcf3, as well as a distinct Lrig1-expressing region above the prospective bulge from which sebaceous glands subsequently emerge (Fig. 2) (Nowak et al., 2008; Jensen et al., 2009; Frances and Niemann, 2012). Other stem cell markers such as Plet1 (recognised by antibody MTS24) and CD34 are not expressed until after sebaceous gland formation and the first completed hair cycle, respectively (Watt and Jensen, 2009; Frances and Niemann, 2012). The outcome from these early developmental events is a patterned PSU with defined compartments demarcated by markers of the future stem cell niches.

Extensive cellular heterogeneity exists within the mature PSU and this has been the topic of a number of excellent recent reviews (Arwert et al., 2012; Rompolas and Greco, 2014; Solanas and Benitah, 2013). Several different populations of cells have been shown to maintain the PSU, and are characterised by the expression of a variety of different markers (see Fig. 1). These include: Lgr5, CD34 and Krt15, which mark the hair follicle bulge stem cells (Liu et al., 2003; Trempus et al., 2003; Jaks et al., 2008); Blimp1 (Prdm1), which marks the sebaceous gland stem cells (Horsley et al., 2006); Gli1 and Lgr6, which mark stem cells in the lower isthmus (Snippert et al., 2010; Brownell et al., 2011); and Lrig1 and Plet1, which mark stem cells in the upper isthmus/JZ (Nijhof et al., 2006; Jensen et al., 2009; Raymond et al., 2010). It is not yet clear whether these different patterns of gene expression reflect specific functions; however, the functional significance of cellular heterogeneity is beginning to emerge from the differences observed in cell behaviour. Several lines of investigation now support a model whereby the epidermis is regionally compartmentalised into functional units that are maintained autonomously (Ghazizadeh and Taichman, 2001; Levy et al., 2005; Page et al., 2013). Here, the lower PSU supports hair follicle growth, while the sebaceous gland, infundibulum and IFE are replenished as independent, yet connected, compartments. This concept opposes the idea that a single population of multipotent stem cells is responsible for epidermal maintenance (Taylor et al., 2000; Lavker et al., 2003, Snippert et al., 2010). In order to understand how long-term homeostasis of the epidermis is maintained, it is necessary to assess distinct cell behaviours within each PSU compartment, as well as their specific requirements for cellular replenishment.

## The PSU and tissue compartmentalisation

The development of improved strategies for transgenesis has enabled the generation of numerous mouse strains that allow temporal and spatial labelling of specific subpopulations of cells in the PSU (reviewed by Kretzschmar and Watt, 2012; Blanpain and Simons, 2013; Alcolea and Jones, 2014). These sophisticated approaches have made it possible to gain new insights into the long-term maintenance of the epithelium and to characterise cellular heterogeneity during steady-state homeostasis, allowing a comprehensive view of how each individual compartment within the PSU is both maintained and replenished.

### The hair follicle

The best-characterised stem cell population in the PSU resides in the bulge region (bulge stem cells; Fig. 1). These cells have been the focus of numerous investigations because of their prominent location, highly quiescent nature (Cotsarelis et al., 1990), extensive clonogenic capacity *in vitro* (Oshima et al., 2001) and their expression of a set of distinct markers (reviewed by Fuchs and Horsley, 2008). Bulge stem cells were initially believed to contribute to the entire epithelium during steady-state homeostasis (Taylor et al., 2000; Lavker et al., 2003). However, fate-mapping experiments have since established that these cells are restricted to lower hair follicle lineages during steady-state homeostasis (Morris et al., 2004; Jaks et al., 2008; Youssef et al., 2010; Page et al., 2013). It has been suggested that hair follicle stem cells contribute to the sebaceous gland (Petersson et al., 2011); however, the minimal *Krt15* promoter used to control Cre expression in these experiments has been associated with ectopic expression in the IFE and the sebaceous gland, providing a false impression of the hair follicle stem cell contribution (Lapouge et al., 2011). The molecular characterisation of bulge stem cells has provided new insights into their biological properties. Importantly, although the overall hair follicle stem cell niche appears homogenous, an underlying cellular heterogeneity exists, including circadian oscillation within the stem cell pool that is important for long-term tissue homeostasis (Janich et al., 2011). Furthermore, the localisation of stem cells within the niche dictates their involvement in hair cycling (Rompolas et al., 2013).

### The IFE and infundibulum

Despite forming a continuous epithelial sheet, the infundibulum is maintained independently from the IFE (Levy et al., 2005; Nowak et al., 2008; Page et al., 2013). Fate-mapping studies in combination with mathematical modelling have proposed various models for tissue maintenance of the IFE. These suggest that IFE maintenance relies either on a combination of largely quiescent stem cells together with proliferating committed progenitors (Mascre et al., 2012) or on a single population of progenitors (Clayton et al., 2007; Doupe et al., 2010; Lim et al., 2013). Importantly, the models were based on four different Cre models and the differing outcomes might reflect the behaviour of distinct populations within the epithelium. Moreover, different epidermal regions – ear, palm and tail epidermis – were studied. The possibility remains that the distinct patterning of the tail epithelium enables the formation of distinct stem cell niches that would not exist in other body parts and that a two-tier system of quiescent stem cells and committed progenitors only operates in restricted areas (Gomez et al., 2013).

Quantitative studies are still lacking for lineage-tracing experiments within the infundibulum. The available data, however, demonstrate that proliferating Lrig1-expressing stem cells located in the upper part of the JZ rapidly replenish the infundibulum (Page et al., 2013; Veniaminova et al., 2013). Interestingly, clones arising in the JZ gradually fill up the infundibulum without contributing to the IFE, suggesting that this compartment is maintained as an autonomous unit (Page et al., 2013). The labelling dynamics imply that transit amplifying cells are rapidly replaced from within the JZ. The requirement for cellular replacement is very likely a reflection of the mechanical stress exerted by movements of the hair, as it is channelled through the infundibulum. Increased shedding of the differentiated barrier within the infundibulum due to this stress is evident by the expression of pro-inflammatory markers in Lrig1-expressing cells. These include β-defensins, which are endogenous antimicrobial peptides, and the monocyte attractants Cxcl12, Ccl2 and Ccl7 (Nagao et al., 2012; Page et al., 2013).

It has been proposed that cells from the PSU participate in the maintenance of the IFE (Morris et al., 2004; Snippert et al., 2010). These conclusions arise from fate-mapping experiments using both *Lgr6* and *Krt15* promoters to drive inducible Cre recombinases. However, it is clear that the ectopic expression activity observed from both of these promoters within the IFE complicates such studies (Lapouge et al., 2011; Snippert et al., 2010; Page et al., 2013; Liao and Nguyen, 2014). The observation that the PSU is maintained independently of the IFE (Levy et al., 2005; Nowak et al., 2008; Brownell et al., 2011; Page et al., 2013) makes it exceedingly difficult to reconcile these apparently contradictory observations, and further investigations are needed to resolve this outstanding question.

### The sebaceous gland

The sebaceous gland plays a very prominent role in lubricating and waterproofing the epidermis. The gland requires constant replenishment to remain functional throughout life, but the identity of sebaceous gland stem cells remains enigmatic. Early data suggested that Blimp1 specifically marked a population of sebaceous gland stem cells (Horsley et al., 2006). However, it was subsequently shown that Blimp1 is more widely expressed in the epidermis, including in differentiated cells of the sebaceous gland, and it has therefore been rejected as a specific marker of sebaceous gland stem cells (Magnusdottir et al., 2007; Cottle et al., 2013). Fate mapping from a minimal *Krt15* promoter and from the *Lgr6* promoter driving Cre expression have led to the conclusion that stem cells residing within the bulge and the lower isthmus replenish the sebaceous gland (Petersson et al., 2011; Snippert et al., 2010). However, the ectopic expression observed from both the *Lgr6* and *Krt15* promoters in the sebaceous gland reinforces the fact that additional studies are required to resolve the issue of whether cells from the bulge and isthmus contribute to sebaceous gland maintenance. Recent lineage-tracing data using Lrig1, which marks basal cells in both the JZ and the sebaceous gland, strongly support the contention that basal cells within the sebaceous gland form an autonomous source for cellular replenishment and that the sebaceous gland is maintained independently of all other compartments (Page et al., 2013).

### Merkel cells

Merkel cells link the epithelium to the nervous system and provide sensory functions. They reside either in enervated regions of the whisker PSU or in touch domes next to PSUs within the IFE (Maricich et al., 2009; Doucet et al., 2013). It was initially believed that this cell type was derived from neural crest, based on its function and expression of neuronal markers; however, conditional knockout, transplantation and lineage-tracing studies have demonstrated an epidermal origin (van Keymeulen et al., 2009; Morrison et al., 2009; Woo et al., 2010). Merkel cells have a slow turnover, which has made analysis exceedingly difficult, but elegant studies involving fate mapping with incorporation of nucleotide analogs have demonstrated that epidermal keratinocytes are responsible for their replacement (van Keymeulen et al., 2009). Whether a specialised subset of cells within the epidermal keratinocyte compartment is responsible for Merkel cell replacement has not yet been addressed.

## Tissue compartmentalisation

The structural composition achieved via epithelial compartmentalisation provides an attractive model for tissue replenishment, since stem cells only need to cater for local cell replacement needs. This design allows compartments within the tissue to undergo renewal at different rates without compromising tissue integrity. This is illustrated by the spatial separation of the highly proliferative JZ and the quiescent bulge region. Interestingly, a tissue compartmentalisation model has recently been proposed for homeostasis of other epithelial tissues such as the prostate, mammary gland and tongue (Van Keymeulen et al., 2011; Choi et al., 2012; Ousset et al., 2012; Tanaka et al., 2013). Such compartmentalisation implies that tissues generally build up seemingly invisible borders between structural elements and that this is relevant for maintaining long-term tissue homeostasis.

The exact molecular mechanisms for maintaining the structural integrity of the individual elements remain unresolved. Insights from elegant developmental studies illustrate that combinations of mechanical forces and cell sorting can form the basis for establishing and maintaining tissue compartmentalisation. Boundaries between different compartments are readily established but their subsequent maintenance requires restricted movement within the tissue. In the developing *Drosophila* wing disc, this is achieved by a zone of non-proliferating cells that forms a boundary between distinct compartments (O'Brochta and Bryant, 1985). Cell sorting has also been associated with the differential expression of adhesion molecules to generate differential adhesion or repulsion between juxtaposing cell populations. Alternatively, the differential expression of secreted signalling molecules can establish distinct cellular identities that preclude disruption of the compartment boundaries (reviewed by Dahmann et al., 2011).

Within the epidermis a number of these mechanisms might work in concert. The quiescent nature of stem cells within the bulge, with respect to both cell division and migration (Cotsarelis et al., 1990; Rompolas et al., 2012), could potentially hinder the downward migration of progeny of highly proliferative stem cells in the JZ, much like the boundary zone formed by non-proliferating cells in the *Drosophila* wing disc (O'Brochta and Bryant, 1985). In a similar manner, balanced cellular replenishment between the IFE and the infundibulum could restrict movement between compartments during steady-state homeostasis. In this case, the structural arrangements of the PSU at an angle to the IFE could create mechanical tension at the junctions between the two compartments and facilitate the formation of a seemingly invisible border. In a more classical manner, the differential expression of adherens molecules such as CD34 and Necl2 (Cadm1) in distinct stem cell populations is likely to affect compartmentalisation (Trempus et al., 2003; Giangreco et al., 2009). The importance of Necl2 in regulating stem cell proliferation is evident, although it is still unknown how loss and gain of Necl2 affect tissue compartmentalisation (Giangreco et al., 2009). The Eph and ephrin family provides a classical example of sorting molecules (Batlle et al., 2002; Solanas et al., 2011); although members are differentially expressed within the epidermis (Genander et al., 2010), their involvement in epidermal compartmentalisation is still unclear. Lastly, the differential expression of growth factor receptors and ligands, as well as extracellular matrix molecules, might endow specific properties within different stem cell niches and thereby contribute to distinct behaviours associated with certain compartments. Bone morphogenetic protein (BMP) receptor signalling represents one such example, as it instructs quiescence in bulge stem cells but drives cellular differentiation within the IFE (Plikus and Chuong, 2008; Ahmed et al., 2011). The extent to which the mechanisms that govern tissue compartmentalisation are reliant on differential proliferative behaviours or the differential expression of signalling and/or adhesion molecules is likely to be resolved by the combination of long-term fate-mapping studies and genetic knockout models. Understanding these mechanisms will provide important insights into how homeostasis is maintained.

## Pilosebaceous stem cells and tissue regeneration

In addition to their important role in homeostasis, stem cells actively engage in tissue repair following injury. The regenerative process is initiated instantly following injury and can be divided into three partly overlapping phases. Initially, injury causes activation of the immune system and initiates the first inflammatory phase. Following the early stimulation of the complement system and bleeding, activated neutrophils, macrophages and lymphocytes enter the wound area to remove pathogens and cellular debris. As the adaptive immune response peaks, keratinocytes enter the proliferative phase of the re-epithelialisation process to regenerate the barrier. Lastly, the regenerated zone, including the underlying dermis, undergoes significant remodelling in order to reinstate tissue contraction and limit scarring. The entire regenerative process is driven by numerous paracrine and autocrine signalling loops between inflammatory cells, fibroblasts and epidermal keratinocytes (reviewed by Gurtner et al., 2008).

In order for stem cell progeny from the PSU to participate in the regenerative response within the IFE, the existing tissue compartmentalisation boundaries need to be broken. Once this has occurred, cells from within the PSU can contribute to both the acute re-epithelialisation and long-term maintenance of the wounded area. The extent of tissue injury, and thus the experimental method used, will very likely influence the regenerative response and the contribution from the PSU. The most commonly employed experimental model for skin injury is full-thickness wounding. This procedure involves taking a biopsy that excises the full thickness of the skin, thereby exposing the underlying muscle facia. Whereas superficial wounds are readily healed by relatively simple processes, full-thickness wounds provide a means to study numerous aspects of regeneration, including stem cell activation. The size of biopsies taken also influences the regenerative response: regenerated epithelium from smaller wounds (<7 mm in diameter) is devoid of hair follicles, whereas larger wounds (>1 cm in diameter) result in *de novo* follicle formation (Ito et al., 2007).

### Tissue regeneration: breaking down the boundaries

In the case of full-thickness wounds, the regenerative process is elicited immediately following injury and, within 24 h, the progeny of stem cells in the JZ are detected in the IFE. The response from bulge hair follicle stem cells is significantly delayed, suggesting that this population might have a different role in the repair process (Page et al., 2013). The fact that PSU-derived cells are detected within the IFE shortly after injury suggests that normal boundaries between the PSU and the IFE do not prohibit cell migration and mixing between compartments following injury (Nowak et al., 2008; Levy et al., 2007). In contrast to full-thickness wounding, cells from within the PSU are not required for tissue repair following incisional wounds in the tail epithelium (Langton et al., 2008), and lineage-tracing data show that IFE-derived progeny in general are a major source for regeneration in response to a small biopsy on the tail (Mascre et al., 2012). It is therefore clear that the severity of the initial trauma greatly influences the regenerative process and the cellular response.

**Fig. 3.**
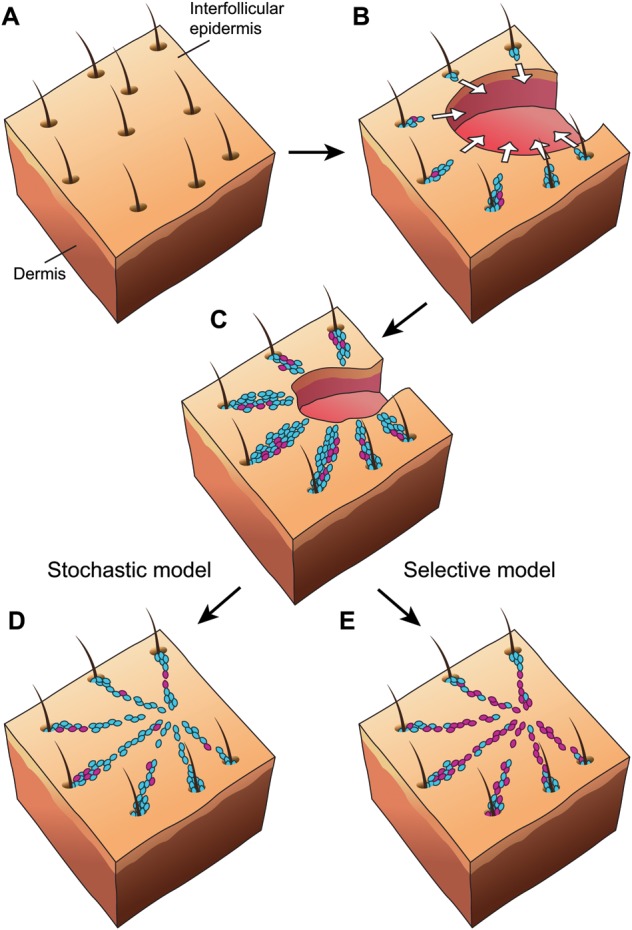
**Mobilisation of stem cell progeny from the PSU following wounding.** Upon full-thickness wounding of intact skin (A), stem cell progeny from multiple PSUs along the wound edge migrate toward the centre of the wound area (white arrows, B) to participate in the regenerative response within the interfollicular epidermis. Two lineage-marked populations represented by blue cells and violet cells are depicted. Re-epithelialisation of the wound area occurs with a larger fraction of blue cell progeny present than violet cell progeny, which can result from the differential timing of stem cell activation or different labelling efficiencies, as shown in C. According to a stochastic model (D), the long-term contribution of stem cell progeny in the wound area is proportional to their initial representation in the wound epidermis. Since there is no difference in the fitness between the blue and violet cells, blue cells outnumber violet cells because of their initial numerical advantage. By contrast, a selective model (E) assumes that intrinsic fitness differences between stem cell progeny do exist. Here, violet cells have an advantage compared with blue cells, so despite being under-represented they eventually dominate the wound area.

Mobilisation of stem cells from the PSU allows them to take part in the regenerative response (Nowak et al., 2008; Levy et al., 2007). Not only the timing of stem cell activation but also the subsequent long-term contribution to the re-epithelialised wound area vary between different populations of stem cells (reviewed by Plikus et al., 2012). For example, the majority of progeny that derive from Krt15-expressing bulge stem cells appear to be rapidly lost following wound healing (Ito et al., 2005), whereas progeny from stem cell populations expressing Sox9, Gli1 and Lrig1 located in the bulge, isthmus and JZ contribute to long-term regeneration (Nowak et al., 2008; Brownell et al., 2011; Page et al., 2013). One of two possible models might therefore describe the regenerative response; a selective model, in which specific stem cell progeny are retained within the regenerated area at the expense of others; or a stochastic model, whereby the fraction of retained stem cell progeny within the regenerated tissue is proportional to their initial contribution (Fig. 3). Although lineage tracing is indispensable for analysing the relative contributions of different cell types during regeneration, one must consider that the end result will inevitably reflect the fraction of labelled cells mobilised to enter the wounded area. Variability in the initial labelling of cells is dependent in part on the experimental setting, but early studies of wound healing did not take this into account (Ito et al., 2005). Interestingly, when normalising fate-mapping data to the initial contribution of PSU-derived cells to the wound epidermis, different PSU stem cell populations contribute in a comparable manner to long-term tissue maintenance (Page et al., 2013). This suggests that cellular identity within the epidermis is very plastic and that the long-term contribution of PSU-derived stem cells to the maintenance of the IFE is not determined by the cellular heritage but appears instead to be a stochastic event (Fig. 3). It remains to be shown whether PSU-derived cells in the IFE are indistinguishable from resident IFE cells or if they have intrinsic properties otherwise associated with their origin in the PSU.

Up until now, it has been argued that bulge stem cells are multipotent based on their ability to be mobilised and enter the IFE upon injury or induction of inflammation (Taylor et al., 2000; Braun et al., 2003; Tumbar et al., 2004). The reverse experiment, in which IFE cells replace damaged PSU compartments, has been difficult to perform due to the inability to efficiently eliminate parts of the PSU (Ito et al., 2005). Elegant lineage-tracing studies using laser ablation of bulge stem cells have recently demonstrated that cells from the upper PSU or the IFE can migrate towards the lower PSU and replace bulge stem cells (Rompolas et al., 2013). Combined with data from skin reconstitution experiments, this illustrates an extraordinary plasticity among epidermal stem cells (Jensen et al., 2009). A model is now emerging whereby tissue-specific stem cells, irrespective of their ancestry, have the potential to contribute to essentially all compartments when provided with an appropriate microenvironment.

### Stem cell activation

Epithelial regeneration is driven by multiple signalling pathways, both paracrine and autocrine, that mediate cell-cell interactions within the wounded area and guide regeneration through the distinct phases (Gurtner et al., 2008). Studies of loss- and gain-of-function mouse models for different signalling networks illustrate the enormous redundancy in the transforming growth factor (TGF) β superfamily, the epidermal growth factor (EGF) and fibroblast growth factor (FGF) receptor family, and in the interleukin and interferon pathways, as only moderate effects are observed when these pathways are modulated during the regenerative process (reviewed by Muller et al., 2012). The hepatocyte growth factor (HGF) pathway, which consists of one ligand and one receptor (HGF and c-Met, respectively), is required during wound repair, and HGF is upregulated very early following trauma (Chmielowiec et al., 2007). As c-Met is highly expressed by various stem cell populations, this might suggest a role in stem cell activation (Chmielowiec et al., 2007). The precise signalling dynamics of the other pathways remains largely unresolved, and it is therefore difficult to assess whether they are involved in stem cell activation and mobilisation or in the subsequent burst of proliferation, or in a combination of these events.

Stem cell activation and mobilisation during normal hair cycling may provide insight into the stem cell response during wounding and regeneration. Hair cycling is initiated by the activation of subsets of hair follicle stem cells by a combination of Wnt, TGFβ and FGF stimulation (Gat et al., 1998; Greco et al., 2009; Oshimori and Fuchs, 2012). Although Wnt promotes hair follicle growth, it is not required for the normal regenerative process (Ito et al., 2005). Members of the TGFβ superfamily have widespread effects on all PSU stem cell populations (Plikus and Chuong, 2008; Oshimori and Fuchs, 2012; Lin and Yang, 2013), and there is evidence to suggest that the inhibitory effects of BMP signalling on wound repair involve misregulation of epidermal stem cells (Lewis et al., 2014). Signalling via basic FGFs is important for PSU maintenance; however, the pronounced inflammatory effects that ensue following loss of the FGF receptors prohibits any meaningful conclusions as to their specific effects on stem cell mobilisation during regeneration (Meyer et al., 2012; Yang et al., 2010). Analysis of signalling networks that instruct stem cells outside the hair follicle bulge will provide additional insight into the mechanisms of tissue repair and tissue compartmentalisation.

## Oncogenic perturbation of stem cell behaviour

Mechanistic insight into stem cell regulation can be gained from targeting specific cancer-associated gain- or loss-of-function mutations to individual stem cell populations. Members of the Ras family of small GTPases are activated downstream of most growth factor receptor pathways and are found to be mutated in a number of different cancers (Schubbert et al., 2007). The expression of constitutively active Ras via its endogenous promoter in cells of interest *in vivo* therefore provides a proxy for constitutively active growth factor receptor activation (Tuveson et al., 2004). Expression of a constitutively active form of Kras in bulge stem cells and the Lrig1-positive stem cell compartment causes increased proliferation specifically within the targeted compartments (White et al., 2011; Page et al., 2013). Studies have also shown that, in certain cases, mice can develop spontaneous papillomas following expression of constitutively active Kras in bulge stem cells. Fate mapping in this case suggests that progeny can traverse the normal boundaries between the PSU compartments and contribute to the sebaceous gland and the infundibulum (Lapouge et al., 2011). Upon mobilisation into the IFE, Lrig1-expressing cells also have the capacity to form papillomas, but only following injury (Page et al., 2013).

Basal cell carcinoma (BCC; see Box 1), which is the most common type of cancer, arises from mutations in the hedgehog signalling pathway (reviewed by Kasper et al., 2012). BCC formation can be efficiently modelled *in vivo* upon loss of patched (Ptch) or expression of a constitutively activated form of smoothened (SmoM2) (Kasper et al., 2012). Modelling BCC induction reveals that certain stem cell compartments are competent whereas other are refractory to tumour formation within their normal niches. Historically, it was believed that BCCs arise from the lower PSU owing to the morphological resemblance of the tumour cells to hair follicles and the expression of hair follicle markers such as Krt17 and Krt19 in tumour tissues (Markey et al., 1992). However, targeting tumour-associated mutations to bulge stem cells only causes local tissue hyperplasia, whereas cells within the IFE readily form invasive carcinomas (Youssef et al., 2010). Dynamic studies illustrate that SmoM2 promotes the reprogramming of IFE cells to a hair follicle fate similar to that observed during development (Youssef et al., 2012). Interestingly, however, it appears that it is not the intrinsic properties of the cells themselves that protects them against tumour formation but rather the surrounding microenvironment, since mobilisation of bulge stem cells carrying BCC-promoting mutations into the IFE is sufficient for rapid tumour induction (Kasper et al., 2011; Wong and Reiter, 2011). Moreover, the observed compartmentalisation is capable of withstanding the pressure of increased proliferation associated with the expression of oncogenes (Youssef et al., 2010; White et al., 2011; Page et al., 2013). This demonstrates that oncogene responses are highly context dependent and suggests that tissue compartmentalisation could represent a mechanism for tumour suppression.

Box 1. **Basal cell carcinoma**

The most frequently occurring cancer in Caucasians is basal cell carcinoma (BCC), which will affect three out of ten people. It is a locally invasive disease that does not metastasise; however, it is still considered malignant as it causes significant destruction of the surrounding tissue and extensive disfigurement. BCC formation is associated with uncontrolled hedgehog (Hh) signalling, and the identification of disease-associated mutations has helped delineate the pathway. Hh signalling is mediated via ligand binding (Sonic, Indian and Desert hedgehog) to the receptor Ptch. This allows Smo to translocate to the primary cilium, where it releases Gli transcription factors from the inhibitor Sufu (Epstein, 2008; Kasper et al., 2012).

Excessive Hh signalling via overexpressed ligands, loss of Ptch, expression of mutant Smo or overexpression of Gli transcription factors in mouse models recapitulates the human disease (reviewed by Kasper et al., 2012). The aetiology of BCC has recently been vigorously debated. Historically, the cell of origin for BCC has been classified as hair follicle stem cells owing to the histological similarity of tumours and hair follicles. Recent lineage tracing does however demonstrate that cells within the IFE are most competent at BCC formation (Youssef et al., 2010). Timecourse resolution of the early events following tumour induction demonstrates that BCC development recapitulates early PSU morphogenesis in a strictly Wnt-dependent manner (Youssef et al., 2012). BCCs therefore provide an excellent *in vivo* example of cellular reprogramming based on the perturbation of a single pathway.

A recent report illustrated that tissue compartmentalisation boundaries within the PSU can be compromised upon perturbation of the Notch pathway (Veniaminova et al., 2013). Notch signalling is governed by the expression of receptors and cognate ligands on neighbouring cells, which forms the basis for lateral signal inhibition, whereby groups of cells organise themselves within a tissue. Epidermal Notch signalling is important for cellular identity and boundary maintenance within the PSU (Pan et al., 2004; Vauclair et al., 2005; Blanpain et al., 2006; Estrach et al., 2006). Notch also represents an important tumour suppressor in the epidermis (Nicolas et al., 2003; Agrawal et al., 2011). In the absence of Notch signalling there is significant inflammatory infiltrate, which could partly explain its tumour suppressor function (Demehri et al., 2012). Inhibition of Notch signalling via expression of dominant-negative GFP-tagged mastermind-like 1 allows stem cell progeny within the upper PSU to migrate into the IFE: an event that is usually prohibited during normal homeostasis (Veniaminova et al., 2013). In light of these observations and the apparent role of inflammation in the mobilisation of stem cell progeny, it is pertinent that future work considers the role of stem cell mobilisation in tissue aging, as evidence points to an important role of inflammation in the loss of stem cell capacity *in vivo* (Doles et al., 2012).

## Compartmentalisation and heterogeneity beyond the epidermis

Stem cell hierarchy models adapted from the hematopoietic system have been utilised to explain tissue maintenance in most tissues. However, such models are not necessarily applicable in a spatially restricted environment such as the epithelia. Indeed, the observed stem cell heterogeneity and plasticity, as well as tissue compartmentalisation, are not exclusive to the epidermis. The compartmentalisation model was originally proposed within the epithelium of the mammary gland, where the luminal and basal cell compartments are maintained as independent entities (van Keymeulen et al., 2011). This model was then subsequently shown to apply to the epithelium of the prostate as well (Choi et al., 2012; Ousset et al., 2012). Although it remains to be shown whether additional tissues are maintained by similar mechanisms, it is tempting to speculate that parts of the stomach, where multiple populations of stem cells have been identified, might be maintained via tissue compartmentalisation (Mills and Shivdasani, 2011; Stange et al., 2013). In the epidermis, the observed compartmentalisation provides a functional explanation of the observed stem cell heterogeneity and the need for cellular replacement (Page et al., 2013), and thus it is conceivable that other tissues might show the same requirements.

## Conclusions

Over the years, a number of models have been proposed to explain epidermal maintenance. These range from the existence of a single common multipotent stem cell population to no apparent stem cells at all. However, recent evidence provides a more comprehensive view that encompasses different functional compartments of the epidermis. Here, multiple discrete stem cell populations with restricted lineage potential under homeostatic conditions serve to maintain specific compartments within the epidermis. This provides unprecedented control of cellular turnover in the distinct compartments and might even serve as a means to guard against carcinogenesis.

The epidermis is our first defence against the environment, and as such must be exceedingly versatile in its ability to respond to change. The boundaries that are so tightly maintained during homeostasis are rapidly lost upon injury, suggesting that the behavioural patterns observed during normal homeostasis are endowed by the local microenvironment rather than by intrinsic cellular properties. It is still too early to provide a conclusive answer as to which mechanisms sustain normal compartmentalisation. Nevertheless, indications from cancer models do suggest that individual compartments maintain distinct microenvironments that control the ability to respond to particular oncogenic signalling networks.

A better understanding of how the microenvironment controls stem cell dynamics and tissue compartmentalisation will be important for identifying the specific factors that instruct cell identity and behaviour. Within the skin, interactions between the dermis and epidermis are integral for homeostasis and for the correct expression of stem cell markers within the PSU (Brownell et al., 2011; Liao and Nguyen, 2014); however, the complexity of this interaction is only starting to be unravelled. Recent evidence reveals that the dermis is highly complex and contains multiple distinct lineages (Driskell et al., 2013). Future investigations will shed light on the reciprocal relationship between populations of cells in the epidermis and dermis that control stem cell identity and behaviour, and how tissue compartmentalisation relates to health and disease.
